# Serial transperineal sector prostate biopsies: impact on long-term erectile dysfunction

**DOI:** 10.3332/ecancer.2016.643

**Published:** 2016-05-23

**Authors:** James JY Chong, Mieke Van Hemelrijck, Declan Cahill, Janette Kinsella

**Affiliations:** 1 Faculty of Life Sciences and Medicine, King’s College London, London SE1 1UL, UK; 2 Division of Cancer Studies, Cancer Epidemiology Group, Research Oncology, King’s College London, London SE1 9RT, UK; 3 Department of Urology, The Royal Marsden NHS Foundation Trust, London SW3 6JJ, UK

**Keywords:** prostatic neoplasms, biopsy, erectile dysfunction, urologic surgical procedures

## Abstract

We wanted to determine whether serial transperineal sector prostate biopsies have a long-term effect on erectile dysfunction (ED). A total of 64 men with prostate cancer entered our active surveillance (AS) programme after a transrectal prostate biopsy as well as a confirmatory initial transperineal sector prostate biopsy (TPSBx). A repeat TPSBx was performed 24 months later as part of our active surveillance protocol. The International Index of Erectile Function-5 (IIEF-5) questionnaire assessed ED at baseline prior to each TPSBx, and at one, three, and six months after first and second TPSBx.

There was a significant short-term deterioration in erectile function on mean IIEF-5 score between baseline (19.5), when compared to one month (10.5) (P <0.001) and three months (18.7) (P = 0.001) following first TPSBx. This resolved at six month follow-up (19.6) (P = 0.681). Following second TPSBx, there was a deterioration in erectile function between baseline (16.6), compared to one month (7.3), three months (13.8), and six months (15.9) (P <0.05) following second TPSBx. Initial TPSBx caused significant short-term ED, which resolved by six months. Serial TPSBx appears to have an adverse impact on erectile function in men monitored on AS, increasing the risk of long-term ED. This risk should be highlighted and discussed during the consent process.

## Introduction

More men with newly diagnosed prostate cancer are opting to enter AS programmes which allows them to defer or avoid radical therapy if their disease is low-risk [1, 2] or until their disease profile changes. This delays the potential complications associated with treatment such as erectile dysfunction (ED). Unfortunately, some of these complications can also arise after biopsy. Prostate biopsy is used in AS to initially risk stratify men and also to serially detect any increase in volume or grade of disease.

Reports on serial transrectal prostate biopsy (TRBx) disagree on whether serial biopsies affect erectile function in men on AS [3, 4]. Transperineal sector prostate biopsy (TPSBx) helps to diagnose a more significant and previously undiagnosed cancer as compared to TRBx [5–7], and in many UK centres it is now a routine part of AS. Short-term complications of TPSBx typically include haematuria, acute urinary retention, and dysuria [5, 8]. There have been relatively few reports on ED after serial sector biopsies performed via the transperineal route [9].

As men are likely to have more than one TPSBx during their AS monitoring, it is important to ensure that possible long-term complications of AS are accurately identified. We wanted here to determine whether serial TPSBx have a long-term effect on ED.

## Materials and methods

The current study consists of a consecutive series of 64 men who underwent TRBx followed by confirmatory TPSBx for entry into our AS programme (September 2009 to September 2010). These same men underwent a further staged TPSBx 24 months later, unless if there were clinical reasons to perform biopsy earlier such as a rising PSA. As part of our AS programme, these men were monitored with six monthly PSA, annual DRE, and a TPSBx every 24 months. TPSBx was performed under general anaesthesia with antibiotic prophylaxis. Men were placed in the lithotomy position and the scrotum was displaced upwards to expose the perineum. Transrectal ultrasound and a brachytherapy template were used to preferentially target the peripheral zone with core biopsies taken systematically on both sides from the anterior, mid, posterior, and basal sectors [5]. Around 24–38 cores were taken depending on prostate volume. A total of 24 cores were taken if prostate volume was <30mL, four from the anterior, mid and posterior sectors. If the prostate volume was between 30–50mL, four additional cores were taken from the basal sector. Around 38 cores were taken if prostate volume was >50mL, five from the anterior, mid, and posterior sectors, and four from the basal sector. A single practitioner performed all biopsies. Pathological characteristics, biopsy time, and complication data were collected from medical records. Erectile function was assessed with the International Index of Erectile Function-5 (IIEF-5) questionnaire prior to each biopsy and then at one, three, and six months following both initial and subsequent TPSBx. The degree of ED was stratified into five groups, based on IIEF-5 score [10]. Men were classified with ED if IIEF-5 score was less than 22. Two men were excluded from analysis of IIEF-5 as they reported no sexual activity (IIEF-5 = 0).

Paired t-test were performed to evaluate difference in IIEF-5. All statistical analyses were performed using SAS 9.4.

## Results

Patients’ demographics and biopsy Gleason scores are summarised in Table 1. Mean age at baseline TPSBx was 64.8 years (±5.9) and mean PSA at baseline was 9.2 (±6.3). The majority of men (52%) presented with T2 disease. The most common Gleason score at first TPSBx was 3+3 (36%), 3+4 (25%), 4+3/4+5 (9%), and benign (30%). At second TPSBx, Gleason scores were 3+3 (25%), 3+4 (11%), 4+3/4+4/4+5 (17%), and benign (45%). Table 2 stratifies the degree of ED at baseline and postbiopsy.

With the first TPSBx, there was a statistically significant difference between the IIEF-5 score pre-TPSBx and one month post-TPSBx (Table 3, P <0.001). Moreover, there was a statistically significant difference in IIEF-5 score between pre-TPSBx and three months post-TPSBx (P = 0.001). At six months post-TPSBx, there was no statistically significant difference anymore with the IIEF-5 score pre-TPSBx (P = 0.681).

With the second TPSBx, there was a statistically significant difference between the IIEF-5 score pre-TPSBx and at one, three, and six months post-TPSBx (Table 3, P <0.05). There was a statistically significant difference in IIEF-5 score difference between the two biopsies, at three and six months post-TPSBx (Table 4, P <0.05).

Of those men with normal erectile function before the initial TPSBx (IIEF-5 22-25), three out of 29 (10%) reported significant deterioration in their erectile function (IIEF-5 <17) six months post-second TPSBx.

Complications with TPSBx were rare. There was no clot retention reported after the first TPSBx. Five men (8%) experienced an episode of clot retention requiring catheterisation following the second TPSBx, with four of these related to urinary tract infection. There was a single episode of acute urinary retention following the first TPSBx, and there were no episodes of sepsis requiring admission with either biopsies.

## Discussion

In our cohort of men, there was a significant short-term deterioration in erectile function at one and three months following first TPSBx, compared to pre-TPSBx levels, which resolved at six month follow-up. Following a second biopsy, there was a deterioration in erectile function at one, three, and six months post-TPSBx compared to pre-TPSBx levels.

The effect of serial TRBx on erectile function remains unclear. Fujita *et al* reported on a study with 152 men on AS. There was a significant reduction in SHIM (an analogue of the IIEF-5) score with serial TRBx, especially after three biopsies [4]. There was also a significant difference in men without ED at baseline progressing to ED at follow-up, mean 3.2 (±2.3) years later. Braun *et al* demonstrated a small reduction in IIEF-6 score in a cohort undergoing serial TRBx [3]. They concluded this change was partially attributable to age-related ED, and the length of time their men were on AS rather than on serial TRBx. Both studies investigated the association between erectile function and biopsy over several years, but did not report on effects in the short and medium-term immediately post-TRBx. Their findings are likely to represent multiple variables influencing erectile function, rather than just the effect of a prostate biopsy.

The significant short-term deterioration in erectile function at one and three months following first TPSBx in our study is similar to that previously reported by Akbal *et al*, which showed a transient increase in ED at one month post-TPSBx that returned to baseline at six months [11]; however this was not seen by Klein *et al*, who reported no significant difference in IIEF-5 scores at one week, one month, and three months post-TPSBx [12]. The mean IIEF-5 score in their cohort was 15.18 which is several points lower than both our group and the Akbal *et al* study, and this in turn may have influenced outcomes.

Pepe *et al* reported on erectile function in 100 men who underwent repeated TPSBx, and concluded that serial TPSBx did not significantly worsen erectile function in the short to medium-term [9]. In contrast, our data suggests that serial TPSBx increases the risk of ED at one, three, and six months postbiopsy. Their study failed to report on erectile function before and after initial TPSBx, and also the time difference between initial and subsequent TPSBx.

The cause of ED postbiopsy is likely to be multifactorial; a combination of organic aetiology and psychological factors. Serial TPSBx involve repeated invasive procedures, and this increases the likelihood of trauma and long-term damage. Zisman *et al* suggested that biopsy related ED may be because of neurovascular bundle damage or secondary trauma because of nerve compression from haematoma or oedema [13]. This has been supported by a recent study from Tuncel *et al* that demonstrated radiological changes in the prostate parenchyma and neurovascular bundle in men with ED postbiopsy [14]. In addition, periprostatic nerve block (PPNB) is a commonly used form of analgesia for prostate biopsy, and it is likely to be partly responsible for neurovascular bundle damage. PPNB is generally believed to be a possible cause of transient ED postbiopsy [15].

The psychological impact of requiring a biopsy, anxiety surrounding a prostate cancer diagnosis, waiting for disclosure of results, and reduced quality-of-life caused by short-term complications of biopsy are all likely to affect erectile function. Zisman *et al* reported that biopsy-related anxiety peaked just before men were given their results, and the anxiety continued at one month postbiopsy in 64% of confirmed prostate cancer diagnosis [13]. Wade *et al* reported that men who complained of postbiopsy complications such as pain, haematuria, and haematospermia as a moderate/major problem had much higher levels of anxiety at one week postbiopsy compared to those who reported symptoms as minor/not problematic [16]. At 35 days postbiopsy, this anxiety was noticeably reduced. This decrease in anxiety over time may partially explain why IIEF-5 scores in our men recover towards prebiopsy baseline between one and three months follow-up. Anxiety and distress are generally not perceived to be associated with management of prostate cancer with AS, [17, 18] and the levels of both are generally favourable compared to anxiety and distress linked to radical treatment [19].

The Massachusetts Male Ageing Study (MMAS) and the Florey Adelaide Male Ageing Study both highlighted incidence of ED increasing with age [20, 21], with the MMAS reporting 26 cases per 1000 men annually in a cohort of men aged 40–69 years, an age range likely to mirror a typical AS group. A strength of our study was therefore to limit the effect of age by assessing erectile function immediately before and after TPSBx, rather than measuring erectile function at baseline and at a fixed follow-up point. Better data on the incidence of comorbidities in our cohort, especially given the strong correlation between LUTS and ED [22] would have helped to distinguish men who may have been predisposed to ED because of their pre-existing comorbidities. Our data was collected immediately before and after each TPSBx to ensure that any potential ED was likely to be linked solely to the TPSBx, limiting the influence of pre-existing comorbidities.

## Conclusion

Our AS protocol specifies a TPSBx every 24 months. An initial TPSBx can cause significant short-term ED, which generally recovers. Our study has shown that there is an increased risk of long-term ED with repeated TPSBx, and this should be considered against disease risk when considering further TPSBx.

The evidence for prostate magnetic resonance imaging (MRI) in selection and monitoring of patients for AS is increasing [23–25], and the use of serial imaging, PSA, and DRE to quantify possible disease progression may be helpful in the consideration of further TPSBx.

This risk of ED should be discussed and highlighted in the consent process.

## Conflicts of interest

The author(s) declare that they have no conflicts of interest.

## Figures and Tables

**Table 1.** Patient characteristics (N = 64).

	N (%)
**Mean Age,** years (SD)	63.4 (5.9)
**Mean PSA**, ng/mL (SD)	9.2 (6.3)
**DRE**	
T1c	23 (35.9)
T2	33 (51.6)
T3	8 (12.5)
**Biopsy 1 Gleason Score**	
Benign	19 (29.7)
3+3	23 (35.9)
3+4	16 (25.0)
4+3	5 (7.8)
4+5	1 (1.6)
**Biopsy 2 Gleason Score**	
Benign	29 (45.3)
3+3	16 (25.0)
3+4	7 (10.9)
4+3	4 (6.3)
4+4	2 (3.1)
4+5	5 (7.8)
Missing	1 (1.6)
**Mean operative time 1**, mins (SD)	4.0 (0.9)
**Mean operative time 2**, mins (SD)	6.0 (1.3)

**Table 2.** Baseline and postbiopsy International Index Erectile Function-5 Score.

	N at pre-TPSBx	N at one month post-TPSBx	N at three months post-TPSBx	N at six months post-TPSBx
**First TP**				
Without ED (IIEF-5 22–25)	29 (45.3%)	0	20 (31.3%)	29 (45.3%)
Mild ED (IIEF-5 17–21)	23 (35.9%)	12 (18.8%)	29 (45.3%)	23 (35.9%)
Mild/Moderate ED (IIEF-5 12–16)	3 (4.7%)	9 (14.1%)	5 (7.8%)	4 (6.3%)
Moderate ED (IIEF-5 8–11)	5 (7.8%)	23 (35.9%)	7 (10.9%)	6 (9.4%)
Severe ED (IIEF-5 1–7)	2 (3.1%)	18 (28.1%)	0	0
No sexual function (IIEF-5 0)	2 (3.1%)	2 (3.1%)	2 (3.1%)	2 (3.1%)
Missing	0	0	1 (1.6%)	0
**Second TP**				
Without ED (IIEF-5 22–25)	13 (20.3%)	3 (4.7%)	5 (7.8%)	12 (18.7%)
Mild ED (IIEF-5 17–21)	27 (42.2%)	5 (7.8%)	25 (39.1%)	24 (37.5%)
Mild/Moderate ED (IIEF-5 12–16)	11 (17.2%)	9 (14.1%)	12 (18.8%)	11 (17.2%)
Moderate ED (IIEF-5 8–11)	5 (7.8%)	7 (10.9%)	4 (6.2%)	8 (12.5%)
Severe ED (IIEF-5 1–7)	3 (4.7%)	25 (39.1%)	4 (6.2%)	1 (1.6%)
No sexual function (IIEF-5 0)	5 (7.8%)	15 (23.4%)	9 (14.1%)	8 (12.5%)
Missing	0	0	5 (7.8%)	0

**Table 3.** Baseline mean and difference in International Index Erectile Function-5 Score.

	Mean (SD)	Mean difference (SD) compared with pre-TPSBx - P-value	Mean difference (SD) compared with previous - P-value
**First TP**			
pre-TPSBx	19.5 (6.11)		
One month post-TPSBx	10.5 (5.4)	9.0 (0.8) <0.001	9.0 (0.8) <0.001
Three months post-TPSBx	18.7 (5.6)	0.8 (1.7) 0.001	-8.2 (6.1) <0.001
Six month post-TPSBx	19.6 (5.4)	-0.1 (1.8) 0.681	-0.9 (1.3) <0.001
**Second TP**			
pre-TPSBx	16.6 (7.1)		
One month post-TPSBx	7.3 (6.9)	9.3 (6.6) <0.001	9.3 (6.6) <0.001
Three months post-TPSBx	13.8 (7.6)	2.9 (3.9) <0.001	-6.6 (6.2) <0.001
Six month post-TPSBx	15.9 (7.7)	0.8 (2.0) 0.003	-2.1 (3.3) <0.001

**Table 4.** Difference in International Index Erectile Function-5 Score between two biopsies.

	Mean difference for first TPSBx (SD)	Mean difference for second TPSBx (SD)	Paired T-test P-value
**Compared to pre-TPSBx**			
One month post-TPSBx	9.0 (0.8)	9.3 (6.6)	0.637
Three months post-TPSBx	0.8 (1.7)	2.9 (3.9)	<0.001
Six months post-TPSBx	-0.1 (1.8)	0.8 (2.0)	0.012
**Compared to previous measurement**			
One month post-TPSBx	9.0 (0.8)	9.3 (6.6)	0.637
Three months post-TPSBx	-8.2 (6.1)	-6.6 (6.2)	0.046
Six months post-TPSBx	-0.9 (1.3)	-2.1 (3.3)	0.011
